# Augmented Anticancer Effects of Cantharidin with Liposomal Encapsulation: In Vitro and In Vivo Evaluation

**DOI:** 10.3390/molecules22071052

**Published:** 2017-06-24

**Authors:** Xue Zhang, Cong-Cong Lin, Wai-Kei-Nickie Chan, Kang-Lun Liu, Zhi-Jun Yang, Hong-Qi Zhang

**Affiliations:** 1 School of Chinese Medicine, Hong Kong Baptist University, Hong Kong, China; zhangxueflora@163.com (X.Z.); 14485680@life.hkbu.edu.hk (C.-C.L.); nickie@hkbu.edu.hk (W.-K.-N.C.); liukanglun@hkbu.edu.hk (K.-L.L.); 2 Changshu Research Institute, Hong Kong Baptist University, Changshu Economic and Technological Development (CETD) Zone, Changshu 215500, China

**Keywords:** cantharidin, anti-proliferative effect, PEGylated liposome, drug delivery system, hepatocellular carcinoma, HepG2, xenograft tumor

## Abstract

PEGylated liposomes have received much attention as pharmaceutical carriers to deliver chemotherapeutic agents for therapeutic purpose. The aim of this study was to prepare and characterize PEGylated liposome of cantharidin and investigate its therapeutic effect on human hepatocellular carcinoma treatment in vitro and in vivo. Liposomal cantharidin was evaluated for their anticancer effects in vitro using human hepatocellular carcinoma HepG2 cells and in vivo using HepG2-bearing nude mice compared to free drug. PEGylated liposome of cantharidin had a particle size of 129.9 nm and a high encapsulation efficacy of approximately 88.9%. The liposomal cantharidin had a higher anti-proliferative effect vis-à-vis free cantharidin in inducing G2/M cell cycle arrest and apoptosis. Liposomal cantharidin killed more HepG2 cancer cells at the same concentration equivalent to free cantharidin. Further study in vivo also showed that liposomal cantharidin achieved a higher tumor growth inhibition efficacy than free drug on hepatocellular carcinoma. As our study exhibited enhanced cytotoxicity against HepG2 cells and augmented tumor inhibitory effects in vivo, the results validate the potential value of cantharidin-liposome in improving the therapeutic efficacy of cantharidin for liver cancer.

## 1. Introduction

Hepatocellular carcinoma (HCC), accounting for 70%–90% of all primary liver cancers, is the third leading cause of cancer-related death worldwide [1]. The disease burden of HCC is increasing worldwide, in terms of both incidence and mortality [2]. The high mortality rate is largely due to lack of effective chemotherapeutic agents with high efficacy and low toxicity.

**Figure 1:**
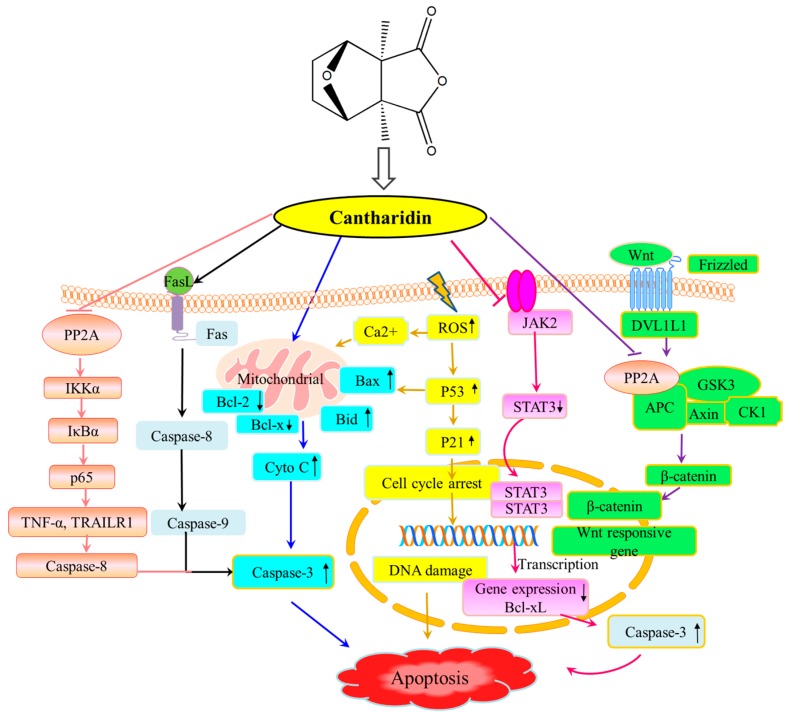
Cantharidin chemical structure and it possible anticancer mechanism. Cantharidin-induced apoptosis is probably via six pathways according to the literature: (a) tumor suppressors p53 and p21; (b) the mitochondrial Bax and Bcl-2 proteins; (c) the JAK/STAT pathway; (d) the transcription factor nuclear factor-κB (NF-κB); (e) Wnt-*β* catenin; and (f) down-regulated. FAS ligand gene (FASLG). Abbreviations: PP2A, protein phosphatase 2A; IKK, IκB kinase; IκB, inhibitor of NF-κB; TNF-α, tumor necrosis factor α; TRAIL, tumor necrosis factor-related apoptosis-inducing ligand; FasL, Fas ligand; Cyto C, cytochrome c; ROS, reactive oxygen species; JAK/STAT, janus tyrosine kinase/signal transducers and activators of transcription; Wnt, wingless-type MMTV integration site family; DVL1L1, dishevelled; GSK3, glycogen synthase kinase 3; APC, adenomatous polyposis coli; CK1, casein kinase 1;

A considerable portion of anti-cancer agents currently used in the clinic are of natural origin [3]. In the quest for natural anti-HCC agents with excellent efficacies for killing cancerous cells, cantharidin drew our attention as a potent chemotherapeutic agent since it has been used as one of many natural products in traditional oriental medicine for cancer treatment [4]. Previous studies have demonstrated this agent with great capacity to induce apoptosis in cancer cells [5,6,7,8,9,10] (Figure 1). In recent years, accumulating experimental evidence indicates that cantharidin holds an anti-cancer property in various types of cancer, such as lung [11], gastric [12], colorectal [13], pancreatic [14] and bladder cancers [6]. Clinical data also show its effects on liver cancer [15].

Apart from the described anti-cancer effects, cantharidin also shows the merit of less drug resistance, which is particularly important when multiple drug resistance (MDR) is a common problem in anti-cancer treatment. Cantharidin has been reported to reverse MDR effectively by down-regulation of P-glycoprotein expression [16], and decreasing expression of MDR-associated gene expression of adenosine triphosphate-binding cassette (ABC)A3 and multi-specific organic anion transporter-B (MOAT-B) [17].

Nevertheless, despite its anti-cancer potency, cantharidin is practically hard to use clinically for liver cancer treatment in its “naked” form, since it has a low accessibility to solid tumor [18]. The effective anti-cancer dose is always associated with a high toxicity to the body causing severe side effects [19].

Modern technology in drug delivery offers a new arena to better utilize traditional medicines and it can hopefully improve the efficacy of natural anti-cancer drugs such as cantharidin [20]. In this connection, liposomes have many advantages to be used as an effective drug delivery system (DDS), such as improving drug solubility and reducing nonspecific side effects and toxicity by encapsulation of the chemotherapeutic molecules [21,22]. With certain surface modification, this new DDS may achieve targeted drug delivery to specific organ or cells to further improve the efficacy and anti-cancer therapeutic index [21,23]. In addition, liposomes are convenient for modification with various ligands for targeted treatment. Polyethylene glycol (PEG)-modified liposomes offers such an advantage of biological stability and extending blood-circulation time by avoiding the destruction by the mononuclear phagocyte system [24].

In this study we attempted to assemble a good DDS for liver cancer treatment by encapsulating cantharidin into PEGylated liposome by testing on human HCC HepG2 cells as compared with “naked” and liposome-loaded cantharidin in vitro by MTT assay, cell cycle analysis and apoptosis study, as well as in vivo tumor growth inhibition efficacy, as our first step in developing an effective DDS for liver cancer.

## 2. Results and discussion

### 2.1. Preparation of Liposomal Cantharidin and Its Characterization

**Figure 2:**
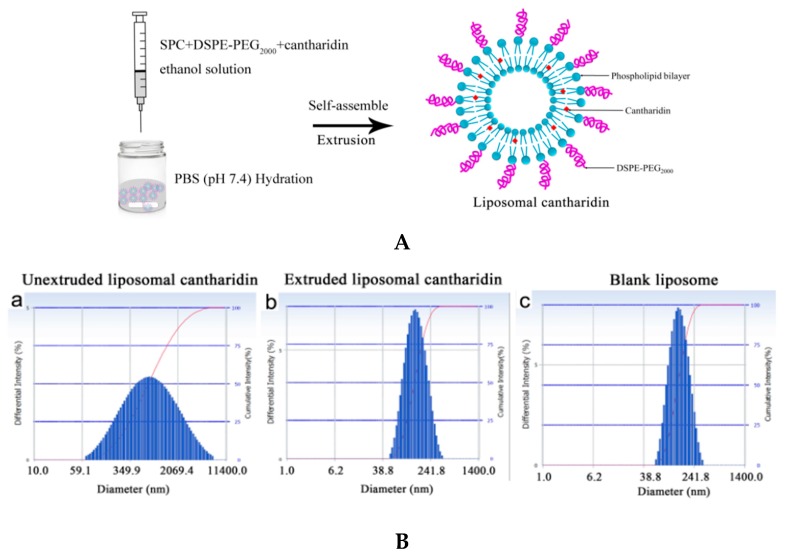
(**A**) Schematic illustration of cantharidin encapsulated into PEGylated liposome; and (**B**) size distribution of liposomes. Particle size of liposomal cantharidin before extrusion, after extrusion, and the blank liposome after extrusion was determined by a Delsa Nano equipment.

This study was designed to evaluate the anticancer activities of cantharidin and liposomal cantharidin for HCC treatment. We firstly prepared liposomal cantharidin by ethanol injection method (Figure 2A). After rapid injection of the lipid carrying ethanol into excess aqueous phase, the lipid particles spontaneously form bilayer vesicles due to the unstable condition [25]. Liposomes prepared in this study appeared as a light milky-white and semi-translucent suspension. The average size of liposomal cantharidin without extrusion showed a larger particle size and a much higher PDI than the liposomal cantharidin with extrusion (Figure 2B and Table 1).

As enhanced permeability and retention (EPR) due to the leaky vascular architecture and the impaired lymphatic drainage is the key factor in advancing the liposomal platform technology [26], and liposomes with particle size controlled at around 100 nm in diameter facilitates extravasation and drug accumulation in tumors via the EPR effect [27]. In this experiment, we utilized a commercial Lipex extruder (Vancouver, BC, Canada) to homogenize liposomal cantharidin to reduce the size and number of lamellae of multilamellar vesicles to unilamellar vesicles [28]. We also managed to obtain liposomal cantharidin with a narrow size distribution peaked at 129.9 nm (Figure 2B) that would favor cancer treatment in vivo. Nevertheless, the encapsulation of cantharidin did not affect the liposomes size, as there was no significant alteration of particle size (~130 nm) of liposomes after encapsulation of cantharidin (Figure 2B).

### 2.2. Anti-Proliferative Effects of Liposomal Cantharidin on HepG2 Cells

In order to evaluate and compare the anticancer effects of cantharidin with or without encapsulation into liposomes, we conducted a series of experiments. 

**Figure 3:**
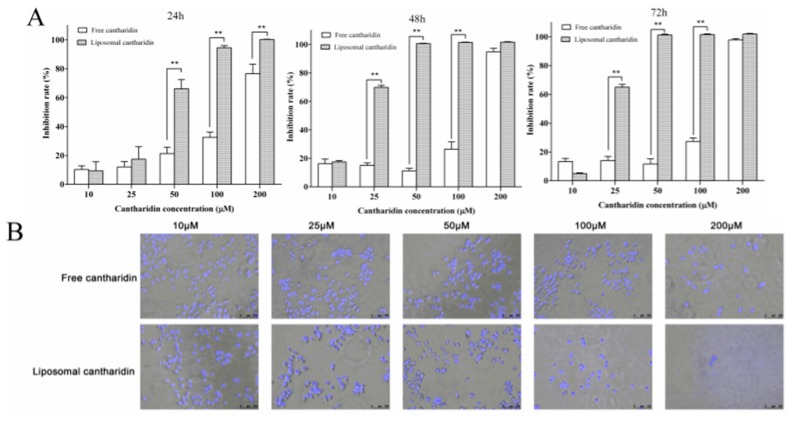
(**A**) Anti-proliferative effects of cantharidin and liposomal cantharidin on HepG2 cells. HepG2 cells were treated with different concentrations of cantharidin and liposomal cantharidin for 24, 48, and 72 h. Results obtained from MTT assay are expressed as percentage of cell growth relative to controls. Results are an average of triplicate experiments and the SD is shown in a bar. ** *p* < 0.01 when compared to the free cantharidin group at the same concentration. (**B**) Morphological observation of HepG2 cells in different treatment groups. HepG2 cells were treated with different concentrations of free cantharidin or liposomal cantharidin for 24 h and stained with Hoechst 33342 followed observation with a fluorescence microscope. The morphological changes of the nuclei of HepG2 cells including chromatin condensation or fragmentation could be seen after treatment with cantharidin and liposomal cantharidin. Scale bar = 100 μm.

Firstly, we examined the anti-proliferative effects of cantharidin and liposomal cantharidin on HepG2 cells. As shown in Figure 3A, the proliferation of cells was apparently suppressed after treatment with free cantharidin and liposomal cantharidin in a dose- and time-dependent manner. However, more importantly, liposomal cantharidin inhibited the growth of HepG2 cells significantly better than free cantharidin (*p* < 0.01), except at extremely high concentration of 200 µM at 48 h and 72 h. In particular, when HepG2 cells were treated with free cantharidin and liposomal cantharidin for 24, 48 and 72 h, liposomal cantharidin inhibited cancer cell growth 3-times, 6.7-times and 5.4-times, respectively, more effectively than the free cantharidin. This suggests that liposome as a drug carrier can augment the anti-proliferative effects of cantharidin in HepG2 cells with relatively low concentration.

### 2.3. Cell and Nuclear Morphology Changes

The anti-proliferative effects of the drugs were further confirmed by morphological examinations under fluorescence microscopy. HepG2 cells treated with cantharidin or liposomal cantharidin with equivalent cantharidin concentration (10–200 μM) were examined and photographed. The cancer cells treated by cantharidin or liposomal cantharidin became shrunken, and round with reduced cell number in a dose-dependent manner (Figure 3B). 

To assess cellular apoptosis, the cell nuclei were stained with Hoechst 33342 and examined as well. The treated cells clearly showed condensed chromatin and nuclear fragmentation, consistent with morphological changes associated with apoptosis, but the cells treated with liposomal cantharidin showed more potent pro-apoptosis activity on HepG2 cells in comparison with cantharidin treatment alone at the equivalent concentration (Figure 3B). 

### 2.4. Cell Cycle Analysis

**Figure 4:**
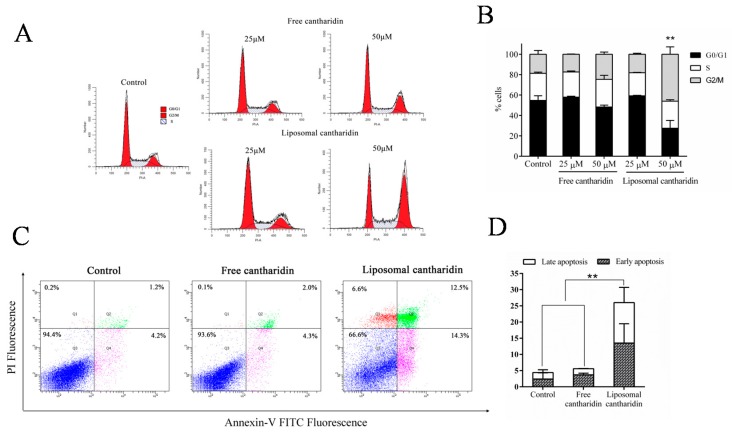
(**A**) Effects of cantharidin and liposomal cantharidin on cell-cycle distribution in HepG2 cells. HepG2 cells were treated with different concentrations of cantharidin and liposomal cantharidin for 24 h and stained with PI. Cellular DNA contents were monitored by flow cytometry. The cell cycle profiles of HepG2 cells under different treatment. (**B**) The percentages of each cell cycle are presented as the mean ± SD of three independent experiments. ** *p* < 0.01 vs. the control. (**C**) Analysis of apoptotic cell death by flow cytometry. HepG2 cells were treated with PBS, 50 µM of free cantharidin, and liposomal cantharidin for 24 h. The apoptotic cell death of HepG2 cells was analyzed with an Annexin V–FITC apoptosis detection kit by flow cytometry. (**D**) The quantitative data for later apoptotic cells(Q2 (Annexin V+ and PI+)) and early apoptotic cells (Q4 (Annexin V+ and PI-)) obtained by flow cytometry (*n* = 3, mean ± SD). ** *p* < 0.01 vs. the control and free cantharidin.

To gain further insight into the mechanism of inhibitory effects of cantharidin and liposomal cantharidin, DNA contents of HepG2 cells treated with cantharidin or liposomal cantharidin for 24 h were analyzed by flow cytometry equipped with Modfit software. Since the cell proliferation profiles (Figure 3) indicate a significantly stronger inhibition at low concentration of liposomal cantharidin, we further tested anticancer effects on G2/M cell cycle arrest in HepG2 cells at 25 and 50 μM. Figure 4A,B shows that cantharidin and liposomal cantharidin caused an accumulation of cells in G2/M phase in a dose-dependent manner, which is associated with the inhibitory effects of cantharidin against HepG2 cells. At the same cantharidin concentration of 50 µM, HepG2 cells were significantly more sensitive to the liposomal cantharidin, once again indicating that encapsulation of the drug into liposome can augment the cantharidin inhibitory effects.

### 2.5. Apoptosis Induced by Liposomal Cantharidin

Since a hallmark of cancers is to avoid apoptosis, the induction of apoptosis in cancer cells is considered crucial in cancer prevention and treatment [29,30]. In this experiment, we detected the cell death of HepG2 cells treated with cantharidin or liposomal cantharidin by means of FITC-Annexin V/PI double staining and flow cytometry assays. The results demonstrated that the percentage of apoptotic cells treated with liposomal cantharidin was significantly increased (*p* < 0.01) in comparison with the control and free cantharidin (Figure 4C,D), indicating once more that the encapsulation of cantharidin into liposome could enhance the drug cytotoxic effects.

### 2.6. In vivo Therapeutic Efficacy

**Figure 5:**
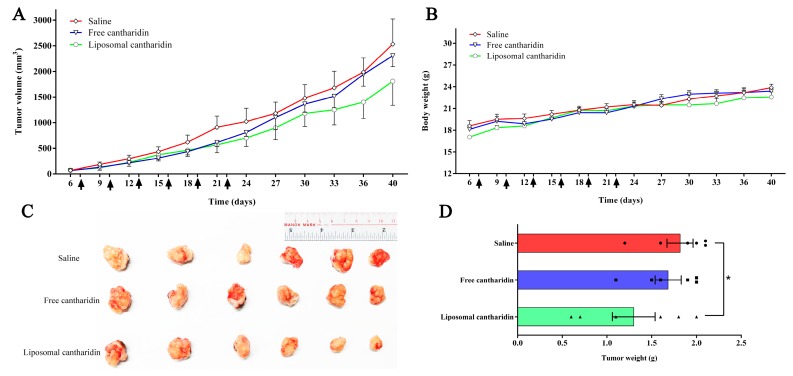
Anticancer efficacy of cantharidin and liposomal cantharidin on HepG2-tumor bearing nude mice in vivo. (**A**) Tumor volume profiles of nude mice in different treatment groups (*n* = 6, mean ± SEM). Mice were administrated with saline, free cantharidin and liposomal cantharidin at a cantharidin dose of 0.35 mg/kg for six intravenous injections in total at three-day intervals. (**B**) Body weights of mice were monitored during the whole experiment periods (*n* = 6, mean ± SEM). (**C**) After 42 days, tumors in different groups were excised and photographed. (**D**) The tumor weight was recorded at the end of the experiments on 42 days. Liposomal cantharidin showed significant lower tumor weights compared to control group (*n* = 6, means ± SEM). * *p* < 0.05 compared to the control.

The HepG2 xenografted in male BALB/c nude mice were used for in vivo therapeutic efficacy study [31]. Liposomal cantharidin and free cantharidin were administrated at 0.35 mg/kg dose of cantharidin by six i.v. injection in three-day intervals. The changes of tumor volumes and the body weights were monitored at three-day intervals for 42 days. As shown in Figure 5A, both cantharidin groups showed tumor inhibition to different degrees, compared to the control group using normal saline. The free cantharidin showed little effect on tumor growth inhibition with the mean tumor volume of 2306.39 ± 214.28 mm^3^ at Day 42. In contrast, the treatment with liposomal cantharidin was more efficacious than that of free cantharidin with a mean tumor size of 1807.35 ± 467.95 mm^3^ (Figure 5A). The in vivo data herewith demonstrated that the encapsulation of the drug into liposome had a better cancer inhibition, probably attributable to a long circulation half-life, and/or better targeting of tumor tissues through the EPR effect, as well as better internalization of the drug by liposome fusion with the plasma membrane to release its cargo into the cytoplasm [32], and/or endocytosis of liposomes by enclosing them into the cancer cell [33]

In the end of experiment the tumor tissues were excited, photographed and weighed ex vivo (Figure 5C). The liposomal cantharidin showed the strongest inhibitory effect on tumor growth with the tumor weight significantly lower than the control group (*p* < 0.05), whereas the free cantharidin did not (Figure 5D).

When the body weight of mice was examined, there was no significant difference between the groups (Figure 5B), indicating that the enhanced cancer inhibition did not come with high systemic toxicity. Above all, the cantharidin encapsulated liposomes had an improved anticancer effects compared to the free cantharidin.

## 3. Materials and Methods

### 3.1. Materials

Cantharidin was purchased from Chengdu Biopurify Phytochemicals Ltd (Sichuan, China); Soy phosphatidylcholine (SPC) was purchased from Taiwei Pharaceutical Co. Ltd (Shanghai, China); 1,2-distearoyl-*sn*-glycero-3-phosphoethanolamine-*N*-[methoxy (polyethylene glycol)-2000] (ammonium salt) (DSPE-PEG_2000_) was purchased from Avanti Polar Lipids Inc. (Alabaster, AL, USA); 3-(4,5-Dimethylthiazol-2-yl)-2,5-Diphenyltetrazolium Bromide (MTT) was purchased from Invitrogen. Dimethyl sulfoxide (DMSO) was purchased from Sigma-Aldrich. Near-infrared (NIR) lipophilic carbocyanine dye 1,1′-dioctadecyltetramethyl indotricarbocyanine iodide (XenoLight DiR) was purchased from Caliper Life Sciences (Hopkinton, MA, USA). Ultrapure water was generated by a Millipore water purification system (EMD Millipore, Billerica, MA, USA). All reagents and chemicals were of analytical grade.

### 3.2. Animals

Balb/c nude male mice (4–5 weeks, 18–20 g) were purchased from Laboratory Animal Services Center, The Chinese University of Hong Kong and acclimatized for 7 days after arrival. Nude mice were housed in individually ventilated cages (IVC cages) of isolated ventilation to avoid microbial contamination. All experimental procedures were done according to guidelines of the Committee on the Use of Human & Animal Subjects in Teaching & Research of Hong Kong Baptist University and the Health Department of the Hong Kong Special Administrative Region. The ethical approval for the project is FRG2/14-15/082.

### 3.3. Preparation of PEGylated Liposome Encapsulating Cantharidin

Liposomal cantharidin (Figure 2A) were prepared using ethanol injection method as described previously [34]. Briefly, cantharidin was encapsulated in liposome containing with SPC and DSPE-PEG_2000_. The mixture of SPC, DSPE-PEG_2000_ and cantharidin (dissolve in ethanol) is rapidly injected into the phosphate buffered saline solution (PBS, pH 7.4) using a syringe needle and stirred for 40 min before extruded through a 0.2 µm pore size filter then 0.1 µm pore size filter (Whatman, Maidstone, Kent, UK) for 5 times sequentially under nitrogen gas using an extruder (Northern Lipids Inc., Burnaby, BC, Canada) to generate unilamellar vesicles of low polydispersity. The encapsulation rate was about 89% as determined by the gas chromatography-mass spectrometer (GC-MS) method. Blank liposome was prepared by the same procedure without cantharidin. 

### 3.4. Drug Analysis

To analyze cantharidin concentration, gas chromatography-mass spectrometry (GC-MS) was performed on a Shimadzu QP-2010 instrument equipped with a Shimadzu AOC-20i auto sampler system and interfaced with a Shimadzu QP 2010S mass spectrometer (Shimadzu Corporation, Tokyo, Japan). Samples were injected in splitless mode on DB-5 MS analytical column (30 m × 0.32 mm ID with a film thickness of 0.25 μm film thickness, J&W Scientific, Folsom, CA). Helium (purity, 99.999%) was used as a carrier gas at a constant flow rate of 1 mL/min. Injector temperature was set at 280 °C. The initial column temperature was 60 °C, and then increased to 275 °C at 10 °C/min, and then to 285 °C at 20 °C/min, held for 3 min. Temperature of the ion source and auxiliary temperature were 250 °C and 285 °C, respectively. Total run time was 40 min. Cantharidin identification was performed by comparison with mass spectra available in NIST 147 library. Calibration curves were linear in the range of 0.1 μg/mL–100 μg/mL (*r^2^* ≥ 0.991).

### 3.5. Characterization of Liposomal Cantharidin

The mean diameter and polydispersity index (PDI) of the prepared liposomes were determined using a Delsa Nano HC Particle Analyzer (Beckman Coulter, Brea, CA, USA) upon dilution with PBS (pH 7.4) used for their preparation to avoid multi-scattering phenomena [35]. Briefly, 0.1 mL liposome suspension was diluted to 1 mL with buffer, and then put into a polystyrene latex cell, and measured with a refractive index of 1.3333 and viscosity value (0.9998 cP) at 20 °C.

### 3.6. Cell Culture

Human HCC HepG2 cells obtained from American Type Culture Collection (ATCC, HB-8065) were maintained in Dulbecco's modified Eagle's medium (DMEM, Gibco Laboratories, NY, USA) supplemented with 10% fetal bovine serum (FBS, Gibco, Grand Island, NY, USA) and 100 U/mL penicillin and 100 μg/mL streptomycin (Invitrogen). Cells were incubated at 37 °C in a humidified atmosphere with 5% CO_2_.

### 3.7. Cell Proliferation Assay

The cytotoxicity activity was determined by the MTT method. The assay is based on the reduction of yellow colored MTT by mitochondrial enzymes in viable cells to purple formazan crystals [36]. Briefly, HepG2 cells were cultured in 96-well plates at a density of 8 × 10^3^ cells/well, and incubated at 37 °C in a 5% CO_2_ incubator. After 24 h, the culture supernatant was replaced with fresh medium or cantharidin free drug and liposomal cantharidin equivalent to the concentration of cantharidin (10, 25, 50, 100, and 200 μM). The plates were incubated for 24, 48, and 72 h. After the exposure period, 20 μL of MTT at a concentration of 5 mg/mL was added to each well. The plates were incubated for 4 h at 37 °C in a 5% CO_2_ incubator. Then, the growth medium was removed and 100 μL of DMSO was added. The plate was covered with tinfoil and agitated on a shaker for 15 min. The absorbance of each well was measured using the Benchmark Plus Microplate Reader (Bio-rad Laboratories) at 570 nm wavelength. Determination of percent of growth inhibition was carried out using the following formula: inhibition rates (%) = ((mean control absorbance-mean experimental absorbance)/mean control absorbance) × 100. The results represented as the average of three independent experiments done over multiple days.

### 3.8. Detection of Apoptosis Morphologically with Hoechst 33342 Staining

To analyze the morphological changes, 1 × 10^5^ cells were incubated in 24-well plates (37 °C, 5% CO_2_). After attachment, the cells were incubated with cantharidin or liposomal cantharidin at 10, 25, 50, 100, 200 µM. After 24 h incubation, the old medium was removed, and the cells were rinsed with PBS. Then, the cells were fixed in 4% paraformaldehyde at room temperature for 5 min, washed and stained with 5 µg/mL of Hoechst 33342 solutions (Eugene, Oregon, OR, USA) in PBS at 37 °C for 10 min. Morphological evaluations of nuclei condensation and fragmentation were performed immediately by means of the fluorescent microscope (Leica, Microsystems, Germany). 

### 3.9. Cell Cycle Analysis

Following the treatments with varying concentrations of cantharidin, cells were subjected to a brief treatment with trypsin, washed twice with cold PBS solution, and re-suspended in 1 mL of 70% ethanol while vortexing. Cells were then fixed with 70% ethanol at 4 °C overnight. Subsequently, the fixed cells were pelleted and resuspended in 1 mL of staining solution containing propidium iodide (PI), RNase and 1X PBS. Fifty thousand fixed cells were analyzed on a FACSCalibur system (BD Biosciences, Franklin Lakes, NJ, USA), with cell cycle profiles analyzed using ModFit program version 3.1 (Verity Software House, Topsham, ME, USA). Samples were run in triplicate and each experiment was repeated three times.

### 3.10. Apoptosis Analysis by Flow Cytometry

FACS analysis of apoptosis was performed utilizing the Annexin-V-fluorescein isothiocyanate/PI (Annexin-V-FITC/PI) Apoptosis Detection Kit I (BD Pharmingen, San Diego, CA, USA) as detailed in the package insert. Briefly, 5 × 10^5^ HepG2 cells were seeded into 60 mm dishes, and allowed to adhere to the plate base over 24 hours. Cells were then treated with medium, PBS, cantharidin free drug and liposomal cantharidin equivalent to the concentration of cantharidin (50 μM) for 24 hours. Following the treatments, the cells were washed with cold PBS (pH = 7.4) twice, and gently re-suspended at 1 × 10^6^ cells/mL in 1X Annexin-V binding buffer. The supernatant (100 μL/tube) was incubated with 5 μL of Annexin-V-FITC and 5 μL of PI for 15 min at room temperature in dark and analyzed by flow cytometry system. The percentage of cells undergoing apoptosis was determined using FACSDiva software (BD Pharmingen, San Diego, CA, USA). At least 20,000 events were recorded for each sample. All experiments were repeated three times. Dual parametric dot plots were then used to calculate the percentage of non-apoptotic viable cells (Annexin V-negative/PI-negative), early apoptotic cells (Annexin V-positive/PI-negative), late apoptotic cells (Annexin V-positive/PI-positive) and mechanically injured cells (Annexin V-negative/PI-positive).

### 3.11. In Vivo Therapeutic Efficacy

In vivo anticancer activity of the liposomal and free cantharidin was evaluated using the subcutaneous HepG2 xenograft tumor model [31]. Briefly, 5 × 10^6^ HepG2 cells in100 μL PBS were subcutaneously injected in the right flank of the male BALB/c nude mice. When the tumor volume reached 50–100 mm^3^ at Day 7 after injection, tumor-bearing mice were randomly divided into three groups (*n* = 6), receiving administration of saline as the control, free cantharidin 0.35 mg/kg, or liposomal cantharidin intravenously, respectively, for six times at a three-day interval. During the experimental period, the tumor volume and body weight were measured every 3 days. The volume of the tumor was calculated using the equation: V = (length × width^2^)/2. At the end of experiment the mice were sacrificed at Day 42, and the tumors were excised, weighed and photographed.

### 3.12. Statistical Analysis

The GraphPad Prism (version 6.01) was used for statistical analyses. The data obtained were presented as means ± standard deviations (SD) for in vitro results and mean ± standard error of the mean (SEM) for in vivo results. Multiple analysis of variance was carried out where necessary. The Dunnett’s multiple comparison test was performed to compare the means between either of the two test groups. Differences were considered to be statistically significant if *p* < 0.05.

## 4. Conclusion

**Figure 6:**
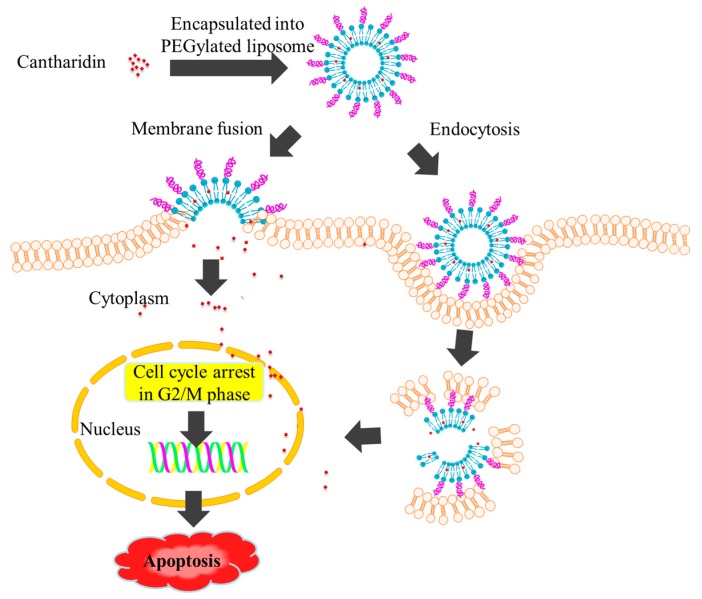
Proposed mechanisms of better anticancer effect of liposomal cantharidin.

Cantharidin encapsulated into liposome composed of SPC and DSPE-PEG_2000_ exhibited an increased anticancer effect in vitro for HCC cells (HepG2) and in vivo for xenografted HepG2 tumor mice vis-à-vis free cantharidin. This better therapeutic effect could be attributable to the longer circulation time and the EPR effect by PEGylated liposomes, as well as better internalization of the drug by liposome fusion with the plasma membrane of the cancer cell (Figure 6). As such, the cantharidin encapsulated into liposomes would be better carried to the target cancer where it is released intracellularly to achieve anticancer effect by inducing apoptosis, offering a new hope for a better treatment of liver cancer.

## Figures and Tables

**Table 1:** Properties of cantharidin-liposome and blank liposome (*n* = 3, mean ± SD)

Liposome Type		Particle Size (nm)	PDI	Encapsulation Efficacy (%)
Liposomal cantharidin	Before extrusion	547.6 ± 2.3	0.329 ± 0.006	
	After extrusion	129.9 ± 2.5	0.087 ± 0.004	88.9 ± 0.1
Blank liposome after extrusion		132.1 ± 0.8	0.071 ± 0.022	
